# Hierarchical strategy for sEMG classification of the hand/wrist gestures and forces of transradial amputees

**DOI:** 10.3389/fnbot.2023.1092006

**Published:** 2023-03-09

**Authors:** Francesca Leone, Federico Mereu, Cosimo Gentile, Francesca Cordella, Emanuele Gruppioni, Loredana Zollo

**Affiliations:** ^1^Advanced Robotics and Human-Centred Technologies, Department at University Campus Bio-Medico of Rome, Rome, Italy; ^2^Istituto Nazionale Assicurazione Infortuni sul Lavoro (INAIL) Prosthetic Center, Vigorso, BO, Italy

**Keywords:** upper limb, prosthetic control, real-time and offline performance, multi-DoFs control, pattern recognition

## Abstract

**Introduction:**

The myoelectric control strategy, based on surface electromyographic signals, has long been used for controlling a prosthetic system with multiple degrees of freedom. Several methods classify gestures and force levels but the simultaneous real-time control of hand/wrist gestures and force levels did not yet reach a satisfactory level of effectiveness.

**Methods:**

In this work, the hierarchical classification approach, already validated on 31 healthy subjects, was adapted for the real-time control of a multi-DoFs prosthetic system on 15 trans-radial amputees. The effectiveness of the hierarchical classification approach was assessed by evaluating both offline and real-time performance using three algorithms: Logistic Regression (LR), Non-linear Logistic Regression (NLR), and Linear Discriminant Analysis (LDA).

**Results:**

The results of this study showed the offline performance of amputees was promising and comparable to healthy subjects, with mean F1 scores of over 90% for the “Hand/wrist gestures classifier” and 95% for the force classifiers, implemented with the three algorithms with features extraction (FE). Another significant finding of this study was the feasibility of using the hierarchical classification strategy for real-time applications, due to its ability to provide a response time of 100 ms while maintaining an average online accuracy of above 90%.

**Discussion:**

A possible solution for real-time control of both hand/wrist gestures and force levels is the combined use of the LR algorithm with FE for the "Hand/wrist gestures classifier", and the NLR with FE for the Spherical and Tip force classifiers.

## 1. Introduction

Hand loss can affect the level of autonomy and the capability of performing the activities of daily life (ADL) for amputees. To remedy this loss, the most used solution is represented by prostheses. Over the years, prosthetic systems have undergone a considerable evolution, both from an aesthetic and a technological/control point of view. Indeed, to date, there are different approaches for prosthetic control but the most widely used is myoelectric control using superficial electrodes for simplicity and versatility. Despite the progress, prostheses are still not natural to control and not very intuitive due to the use of the agonist-antagonist muscle pair, e.g., the flexion-extension of the wrist, for the opening-closing of the prosthetic hand. In Cordella et al. (2016), the real needs of amputees have been analyzed, highlighting which are the most important: performing ADL, having sensory feedback, regulating grip force by lightening the visual attention and the cognitive load for the user, avoiding the sliding of the grasped object (Cohen and Rosenbaum, 2004), and manipulating objects in a fine way. Although current prostheses allow most ADL to be performed, to actively control the grasp force during the execution of a grasping and manipulation task needs force feedback. Some studies have investigated the different solutions, represented by vibrotactile stimulation (Kaczmarek et al., 1991) and electrical stimulation (D'anna et al., 2017; Graczyk et al., 2018; Zollo et al., 2019; Bensmaia et al., 2020). These approaches are in research scope and far from being implemented in commercial solutions. Using sEMG, various PR-based controls have been developed allowing for a higher number of gestures than commercial prostheses (Geethanjali, 2016; Mereu et al., 2021; Latour, 2022). The training procedure includes repeating the gestures at a single intensity but might not be the best solution. Indeed, the use of EMG signals at different intensities for training PR systems can lead to improvements in accuracy and robustness (Scheme and Englehart, 2011; Samuel et al., 2019).

In Al-Timemy et al. (2013), the effect of muscle intensity variations related to the intentional grasp force was investigated on two transradial amputees. The performance of the LDA classifier with time-domain features was compared with autoregression coefficients and a root mean square features set. The performance of the LDA classifier was better with time-domain features and when training it with all force levels, while it degraded up to 60% when the force level varied. In Luppescu et al. (2016), three different classifiers, LDA, the Naïve Bayes, and the multi-class SVM were tested to classify the following: spherical grip, index flexion, hook grip, thumb flexion, fine pinch, and tripod grip. Nine different transradial amputees were asked to perform each gesture with three different force levels (low, medium, and high). The testing accuracy for LDA, Naive Bayes, and the multi-class SVM classifiers, for the six motion classes, was 96.18, 78.65, and 88.76%, respectively. When considering the three force levels applied to each motion gesture (for a total of 18 classes), the accuracy of the above classifiers decreased to 93.11, 76.86, and 86.53%, respectively. In Jabbari et al. (2020), a long short-term memory neural network with the fusion of time-domain descriptors was employed to discriminate six grip gestures at three different force levels (low, medium, and high): 1-thumb flexion, 2-index flexion, 3-fine pinch, 4-tripod grip, 5-hook grip, and 6-spherical grip (power). The results obtained from nine transradial amputees showed the long short-term memory neural network with the fusion of time-domain descriptors set achieved the best average classification errors values (6.4 ± 3.3, 8.6 ± 3.0, and 9.2 ± 5.6% for the low, medium, and high force level testing, respectively).

Other studies (Chen et al., 2013; Irastorza-Landa et al., 2017; Leone et al., 2019; Gentile et al., 2022; Li et al., 2022), employed the hierarchical surface electromyography (sEMG) classification strategy to increase the number of degrees of freedom (DoF) and/or improve the accuracy.

However, the possibility of simultaneously controlling gestures and forces in an intuitive and more natural way, allowing amputees to perform a task with a lower cognitive effort, has not still been investigated. In this study, a hierarchical pattern recognition (PR)-based control was developed allowing the recognition of seven gestures and, for grasping tasks, of three levels of force. This approach is based on the strategy developed by Leone et al. (2019). To apply this strategy to amputee patients, the grasping tasks were performed with both limbs to provide information relating to the force applied with the healthy limb that could be correlated with the muscle activity of the amputated limb, which was used for the classification. In addition, a new method was investigated to make the amputee's perception of the force setting more natural, exploiting the receptors of the musculotendinous junction (Barker, 1967). These receptors receive information directly from the spinal cord and cerebral cortex and increase the sensitivity of the receptor by exciting the intrafusal muscle fibers causing constant control, even during the phases of muscle contraction, and increasing the perception of movement and the position of the same muscle (Liddell and Sherrington, 1924).

The paper is structured as follows: in Section 2, the experimental protocol, the proposed algorithms, and the PR-based architecture are explained; the following section outlines the results obtained for both offline and real-time performance; in the Section 4, the results are examined and a comparison of the proposed method with the literature is presented; and the final section features the conclusions.

## 2. Materials and methods

### 2.1. Experimental setup

**Figure 1:**
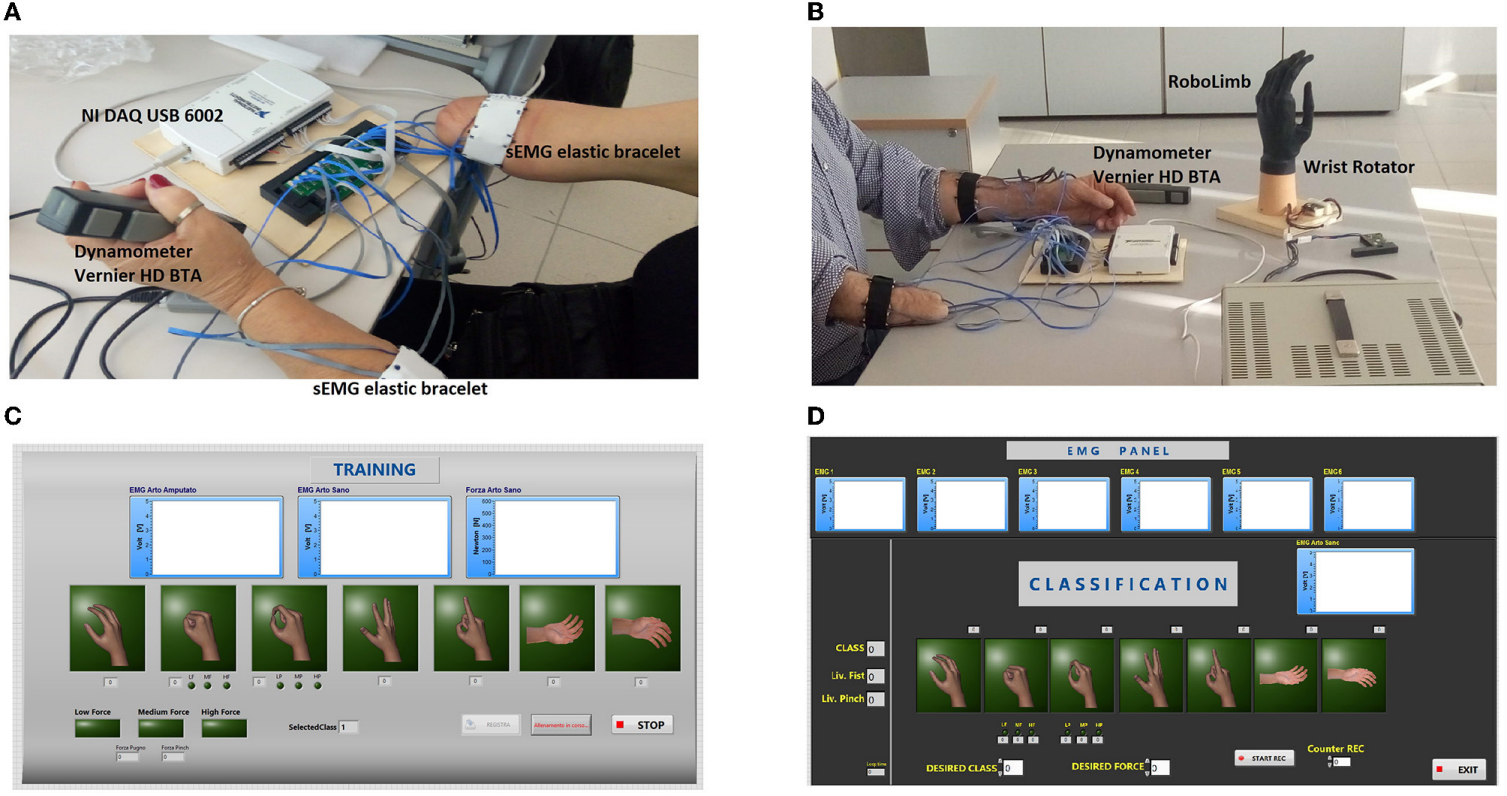
**(A)** The experimental setup for steps 1 and 2. Six sEMG sensors for each elastic bracelet, the NI DAQ USB 6002, and two hand dynamometers. **(B)** The experimental setup for step 3. The same elements of **(A)** with the RoboLimb and wrist module. **(C)** The software interface for step 2. **(D)** The software interface for step 3.

Fifteen transradial patients were enrolled (see Supplementary Table 1 for the patients' information), 12 male and 3 female (aged 45 ± 13.44), at the INAIL prosthesis center in Vigorso di Budrio. All patients gave informed consent^1^ for voluntary participation in the study. The experimental setup was composed of 12 commercial sEMG sensors (Ottobock 13E200 = 50, 27 × 18 × 9.5 mm) and two hand dynamometers (Vernier HD BTA, 46 × 28 × 170 mm), which were used for EMG and force signal acquisition, respectively. A custom electronic interface (Figure 1A) was developed to connect the sensors to the NI-DAQ 6002 (National Instruments). The described experimental setup was connected *via* USB to the PC [MSI prestige 15, Intel (R) Core (TM) i7-1185G7 CPU @ 1.80 GHz 2.40 GHz] to allow data acquisition with *ad-hoc* software developed in LabView. For each arm, six sEMG (Riillo et al., 2014) sensors were located equidistantly from each other on an elastic bracelet and positioned 4 cm below the patient's elbow (Figure 1). For each acquisition, patients were asked to perform gestures with both hands.

Preliminary tests were conducted to correctly define force thresholds and the prosthesis control modality.

- **Force threshold evaluation**
  To identify the three thresholds for the three force levels, two preliminary methods were tested.
  1. The patient was asked to perform the maximum contraction of both limbs; then, the three force thresholds were calculated at 30, 60, and 90% (within a range of ±10%) of the maximum force value, corresponding to the maximum muscle contraction, measured with the hand dynamometer on the healthy limb.
  2. Using the amputee's perception of the force (Barker, 1967), the patient was asked to contract both limbs with three levels of perceived force (low, medium, and high), measured with the dynamometer on the healthy limb.
  Once the three thresholds were defined, in method 1, the patient was provided with visual feedback *via* colored bars (red for high, yellow for medium, and green for low) as to which threshold to perform next. In method 2, no feedback was provided. Amputees had difficulty reaching and maintaining the three thresholds used in method 1, while method 2 proved to be more natural and more repeatable. For this reason, method 2 was used for the determination of the threshold.
- **Prosthesis control**
  The RoboLimb hand is controllable in terms of velocity by setting pulse with modulation (PWM) values (RoboLimb manual). For the movements related to the platform, point, pronation, and supination classes, the PWM values were set at 50%. The validation of the force classifiers needs to measure the grasping force of the prosthesis. As the grasping forces with the RoboLimb cannot be set, tests were performed in which the PWM was varied and the grasping force was measured with the dynamometer. From the tests, the determination of three PWM values (25, 50, and 75%) corresponding to three strength values (approximately 7.5N, 15N, and 30N respectively) was possible.

Then, the experimental protocol provided the following steps:

- **Step 1 - Force-thresholds settings**
  The force signal was acquired through the interface composed of NI-DAQ and a custom board. During the tests, the patient was asked to grasp the hand dynamometer and perform the spherical and tip grips at the three force levels. Three repetitions lasting 3 s were performed for each force level. The mean values obtained from three acquisitions were considered to define the low, medium, and high force levels, for spherical and tip grasps. The low level was fixed between the ± 10% of the low threshold, the medium level was fixed as ±10% of the mean value for the medium threshold, while the high level started from −10% of the highest threshold and continued until the maximum value.
- **Step 2 - Training step**
  The EMG and force signals were acquired through the above-mentioned setup. The patient was asked to perform bi-manually six times and hold each of the following seven hand/wrist gestures for 3 s: rest (hand relaxed), spherical (hand with all fingers closed), tip (hand with thumb and finger touching as if picking a small object), platform (hand completely open and stretched), point (hand with all fingers closed except for the index finger), wrist supination, and wrist pronation (with relaxed hand). For the grasping tasks, the amputees were asked to also modulate the force according to three force levels, as established in Step 1. For each gesture, six repetitions were acquired. The recorded sEMG data were organized in a DataSet matrix (33,600 rows 6 columns). Each column of the matrix was coupled with an EMG sensor, while the rows represented the recorded samples.
- **Step 3 - Online validation**
  A prosthetic system, composed of a hand device (RoboLimb, Touch Bionics) and a wrist module (Wrist Rotator, Ottobock) was employed to evaluate the real-time robustness of the proposed strategy. A custom electronic board and relative firmware were developed to control the prosthesis *via* Bluetooth. The EMG signals were acquired through the interface composed of NI-DAQ and a custom board; the classification of gestures was obtained, as was the classification of force levels for the spherical and tip classes. Moreover, during the performing of each motion class, the outputs of the classifiers were recorded three times for 5 s (Kuiken et al., 2009) (for a total of 33 recordings) to evaluate the real-time robustness of the PR-based system.

**Figure 2:**
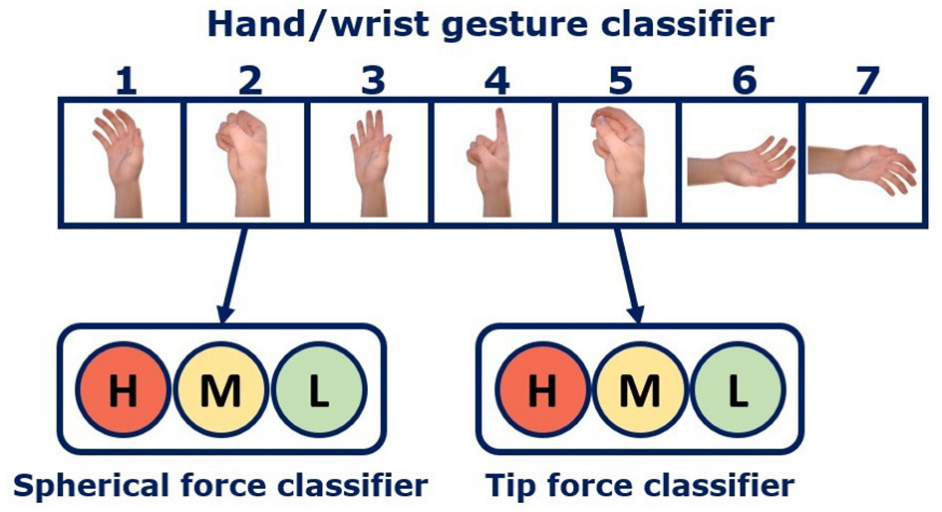
Hierarchical classification strategy description. The “hand/wrist gestures classifier” outputs are represented by these *seven* different gestures: 1, Rest; 2, Spherical; 3, Platform; 4, Point; 5, Tip; 6, Supination; 7, Pronation. If its output class is “spherical”, the “spherical force classifier” will work simultaneously to classify the *three* force levels (H = High, in red; M = Medium, in yellow; L = Low, in green). Likewise for tip.

Three algorithms were used for the classification: logistic regression (LR), non-linear logistic regression (NLR), and linear discriminant analysis (LDA). In particular, LR was also considered, in addition to the NLR algorithm, to simplify the training step of the model (without the polynomial expansion) and speed up the real-time prediction (within 80 ms). For each proposed algorithm, three classifiers were created: the first was employed for gesture recognition, namely “hand/wrist gestures classifier”; while the other two, “spherical force classifier” and “tip force classifier”, could identify three force levels (Leone et al., 2019). If the output of the “hand/wrist gestures classifier” is spherical or tip (Figure 2), the relative force classifier will work simultaneously to classify the force level (low, medium, or high). In particular, the enveloped EMG signal was acquired at 1 kHz, as the specified frequency bandwidth for the sensors is 90–450 Hz (Ottobock, 2008). When the training was performed without FE, the “raw” enveloped EMG signals were used as input to the classifier. For each motion class, 4,800 samples, recorded from each of the six sensors, were used as input for the classification system. Advanced signal processing was not necessary due to the high quality of the EMG signal provided by the Ottobock 13E200 active sensors, which have a common mode rejection ratio of more than 100 dB. The internal circuitry of the sEMG sensors includes an amplification stage and two filters: a high-pass filter to remove motion artifacts and temperature fluctuations, and a low-pass filter to eliminate high-frequency interference from sources such as radio broadcasts, phones, and computers (Ottobock, 2008).

### 2.2. Features extraction

In the literature, a comparative analysis between LDA with time-domain features extraction (FE) and NLR without FE showed no statistically significant difference for the classification performance of five hand gestures (Bellingegni et al., 2017). In this study, the complexity of the classification system was increased by extending the classification from five to seven classes and also considering three different force levels by using the same number of sensors. As the use of EMG PR-based strategies with time-domain features has often been used to improve the robustness of advanced prosthetic systems (Geethanjali and Ray, 2014), the performance of the proposed PR-based control strategy with and without the FE step was verified. A statistical analysis based on the *U*-test with Bonferroni correction (*p* < 0.016) was used for this purpose.

In detail, the following features (Too et al., 2019) were extracted from the “raw” EMG data by using each analysis window of 150 ms with an overlap of 50 ms (Smith et al., 2011): the enhanced mean absolute value, enhanced wavelength, slope sign change, root mean square, and variance. Additional details can be found in Supplementary Section 1 of the Supplementary material.

### 2.3. Logistic regression (LR) and non-linear logistic regression (NLR) classifiers

The LR, or perceptron, is a linear and binary supervised classification algorithm used to come up with a hyperplane in feature space to separate observations belonging or not belonging to a class.

The class membership probability is evaluated using the logistic function (Equation 1) for both LR and NLR classifiers:


(1) $$\begin{array}{l} \;P\left(1|x,\theta \right)=g\left(\theta^{T}\cdot x\right)=\frac{1}{1+e^{-\theta^{T}\cdot x+\theta_{0}}} \end{array}$$

where *g*(·) is the logistic function and θ and θ_0_ are the classification parameters vector and bias term, respectively.

In the linear case (LR), x represents the polynomial features of first grade. For the NLR classifiers, additional polynomial features of x (e.g., $x_{1},x_{2},x_{1}*x_{2},x_{1}^{2},x_{2}^{2}$) were introduced to non-linearize this logistic regression model. Once the optimal model was found, different decision thresholds were tested and the one that maximized the F1 score values was chosen.

The training and test were subdivided by considering the *two ways data split approach* (Ripley, 2007): 70% of the data was reserved for the “*training set”*, while the remaining 30% of the data was reserved for the “*test set”*. The overfitting was mitigated by using a random shuffle to fill these subsets with a proper proportion of all classes' sample distributions. Each single classifier was iteratively trained with all possible configurations of its internal parameters that had an appropriate range of values (Bellingegni et al., 2017). In this way, the bias effects were reduced, and, considering an estimate of the generalizing ability of each classifier, it was possible to explore the best model. For the LR and NLR algorithms, the first-order iterative optimization algorithm “gradient descent” was used to set the optimal internal parameters. A one vs. all approach was introduced to adapt the LR and the NLR classification algorithms to the multi-class classification problem. Additional details can be found in Supplementary Section 2 of the Supplementary material.

### 2.4. Linear discriminant analysis

The LDA is a binary supervised machine learning algorithm and guarantees the maximum class separability (Welling, 2005) by transforming the features into a lower dimensional space, maximizing the ratio of the between-class variance to the within-class variance.

The class label (c) was predicted as follows Equation (2):


(2) $$\begin{array}{l} \begin{array}{c} h_{\beta }\left(x\right)=\max_{c}{(}_{c}\beta^{T}\cdot x{+}_{c}\beta_{0}) \end{array} \end{array}$$

where _*c*_β and _*c*_β_0_ are the classification parameters vector and the bias term of c class, respectively.

Additionally, a random shuffle was implemented to fill these subsets with a proper proportion of all classes' sample distributions. A one vs. all approach was implemented to solve the multi-class classification problem with a binary algorithm as the LDA. The Matlab Classification Learning tool was used to develop *ad-hoc* software for the construction of each of the three LDA classifiers. Additional details can be found in Supplementary Section 3 of the Supplementary material.

To summarize, each of the three algorithms were tested as a classification system. Each classification system was composed of three different classifiers, one for hand/wrist gesture recognition and two for the force levels. Finally, for the real-time analysis, the results of the classification system, for each algorithm, with the FE step to evaluate which one guaranteed better performance, was reported.

## 3. Results

### 3.1. Offline performance

The results of a comparison among the performance of the three algorithms (LR, LDA, and NLR) with or without the feature extraction step were presented. The offline classification performance on 15 transradial amputees was evaluated. For all the classifiers, performance was evaluated in terms of accuracy and F1 score.

**Figure 3:**
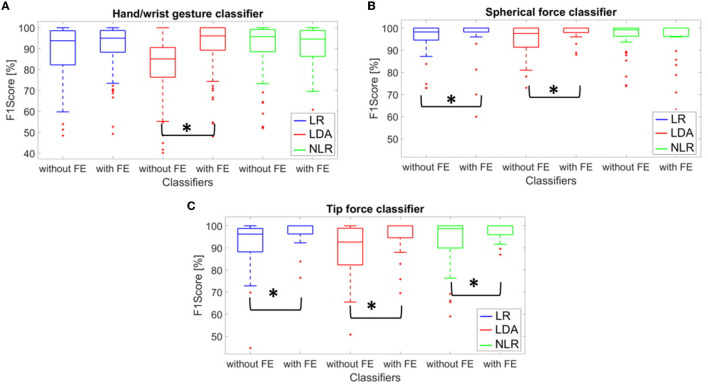
**(A–C)** F1 score mean values calculated for 15 transradial amputees using the LR (in blue), LDA (in red), and NLR (in green) algorithms for the “hand/wrist gestures classifier” **(A)**, the “spherical force classifier” **(B)**, and **(C)** the “tip force classifier” algorithm **(C)**, tested on the “*test set”* with and without the five time-domain FE. Statistical significance is indicated by “*”.

The statistical analysis, based on the *U*-test, was applied to the F1 score values to highlight significant differences in terms of performance with or without the FE, for the three algorithms. The average F1 score values are presented in Figure 3: for the “hand/wrist gesture classifier”, only the LDA algorithm pointed out the statistically significant differences between the aforementioned cases. Instead, for the “spherical force classifier”, both the LR and LDA algorithms showed a statistically significant improvement of the F1 score with the FE. This trend was also confirmed for the “tip force classifier” for the three algorithms. The following results consider the FE.

For the “hand/wrist gesture classifier”, the mean classification accuracy reached the following values: 91.68 ± 2.84, 92.11 ± 2.89, and 90.70 ± 2.77 for the LR, LDA, and NLR algorithms, respectively, (see details in Supplementary Table 2). For the force classifiers, the mean accuracy was above 95% for all three algorithms (see details in Supplementary Table 3).

**Figure 4:**
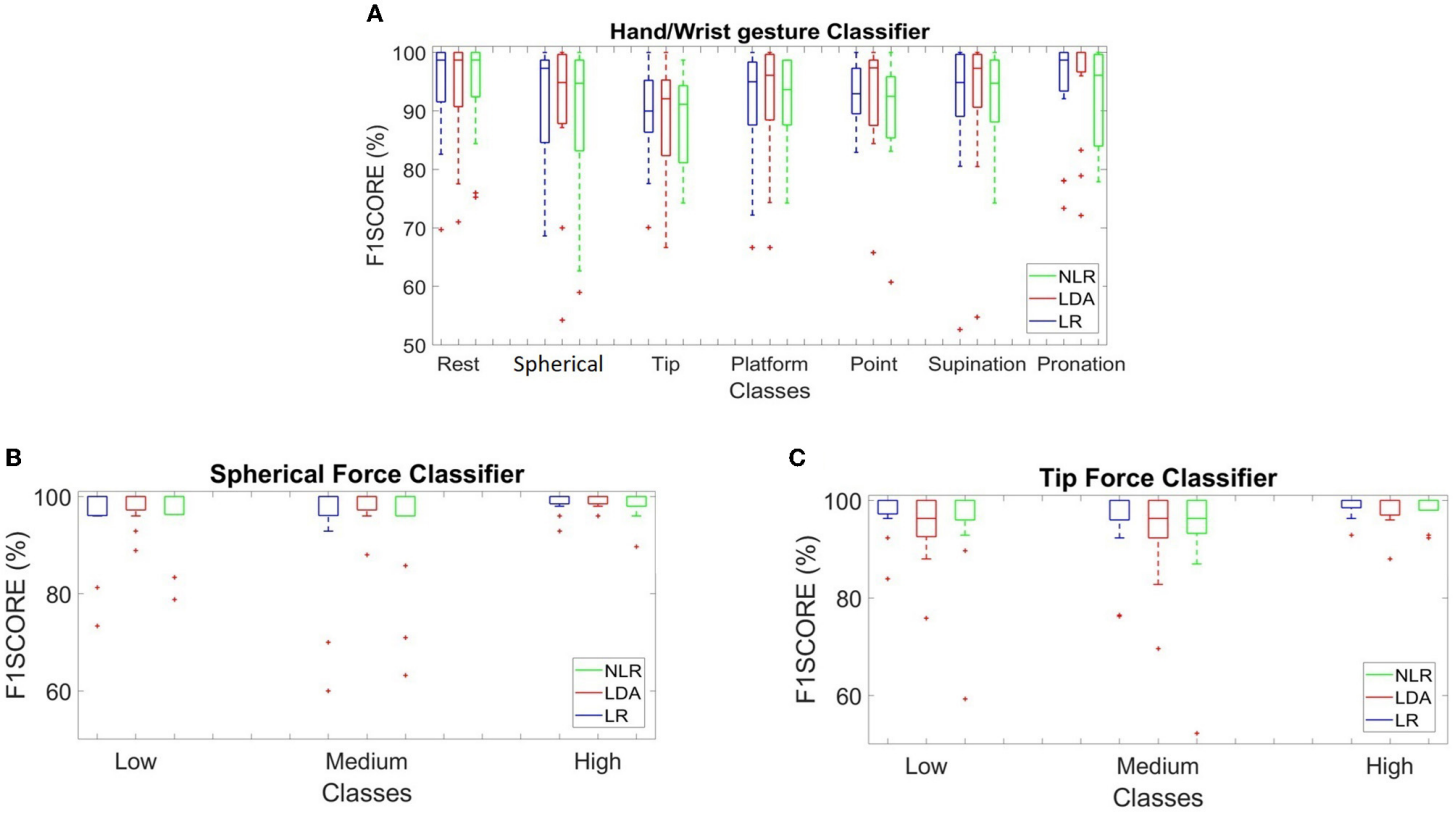
**(A–C)** Average F1 score values calculated for 15 transradial amputees using the LR (in blue), LDA (in red), and NLR (in green) algorithms, tested on the “*test set”* with the five time-domain features extraction (FE) step for the “hand/wrist gestures classifier” (the F1 Score of the seven hand/wrist gestures classes have been reported) **(A)**, the “spherical force classifier” **(B)**, and the “tip force classifier” algorithm **(C)**. The F1Score values of the three force levels have been shown. No statistically significant differences based on a *U*-test with Bonferroni correction among classes were found for the LR, LDA, and NLR classifiers.

The box plots in Figure 4 provide an overview of the F1 score values with FE among the motion classes for the three classifiers. For the “hand/wrist gestures classifier”, the motion classes with the lowest F1 score mean values (approximately 90%) were as follows: the tip class and point class with the LR algorithm; the spherical class and tip class with the LDA algorithm; and the tip class, point class, and wrist supination class with the NLR algorithm. For both the “spherical force classifier” and the “tip force classifier”, the mean F1 score values were above 95 % for the three algorithms. The comparative analysis applied to F1 score values, based on the *U*-test with Bonferroni correction (*p* < 0.016), reported no statistically significant difference among the LR, LDA, and NLR classifiers.

For the three algorithms, the “hand/wrist gestures classifier” showed the highest values of misclassification errors for the spherical and tip grasping tasks. The confusion matrices (in Supplementary Figure 1) confirmed the results of the classification accuracy.

For the “hand/wrist gestures classifier”, the spherical and tip motion classes presented some misclassified data out of the main diagonal. For the “spherical force classifier” and “tip force classifier”, the majority of the misclassified data out of the main diagonal were related to the low and medium force levels.

### 3.2. Real-time performance

A real-time performance evaluation was carried out for each proposed algorithm applied to the hierarchical classification approach. The accuracy of the online classification of the proposed strategy was estimated by asking the subjects to perform the overall movement classes, modulating the three different strength levels for the spherical and tip classes as well. All the tasks were replicated by the prosthetic system, modulating the three levels of force with three different closing speeds of the prosthetic hand.

**Figure 5:**
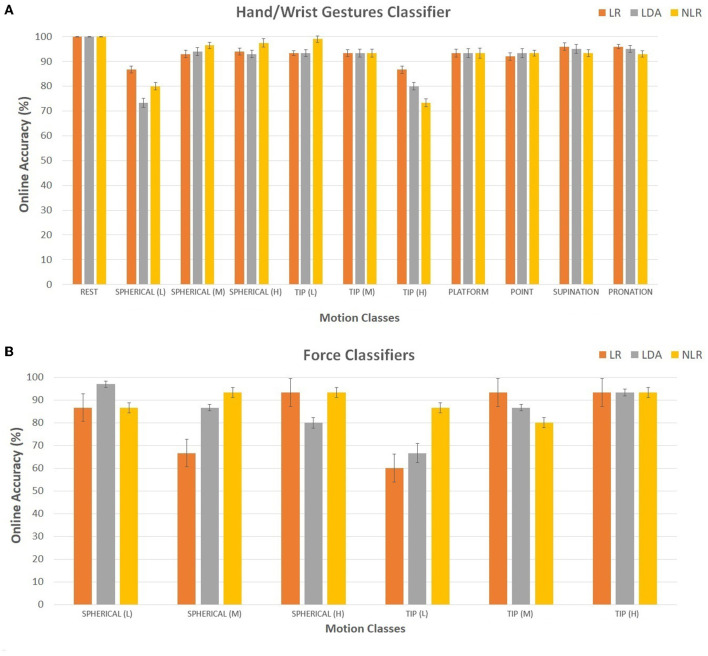
**(A, B)** Online accuracy calculated for 15 transradial amputees using the LR (in orange), LDA (in gray), and NLR (in yellow) “hand/wrist gestures classifier” **(A)** and the LR (in orange), LDA (in gray), and NLR (in yellow) force classifiers **(B)**: the “spherical force classifier” and the “tip force classifier” for the spherical and tip grasp, respectively.

**Figure 6:**
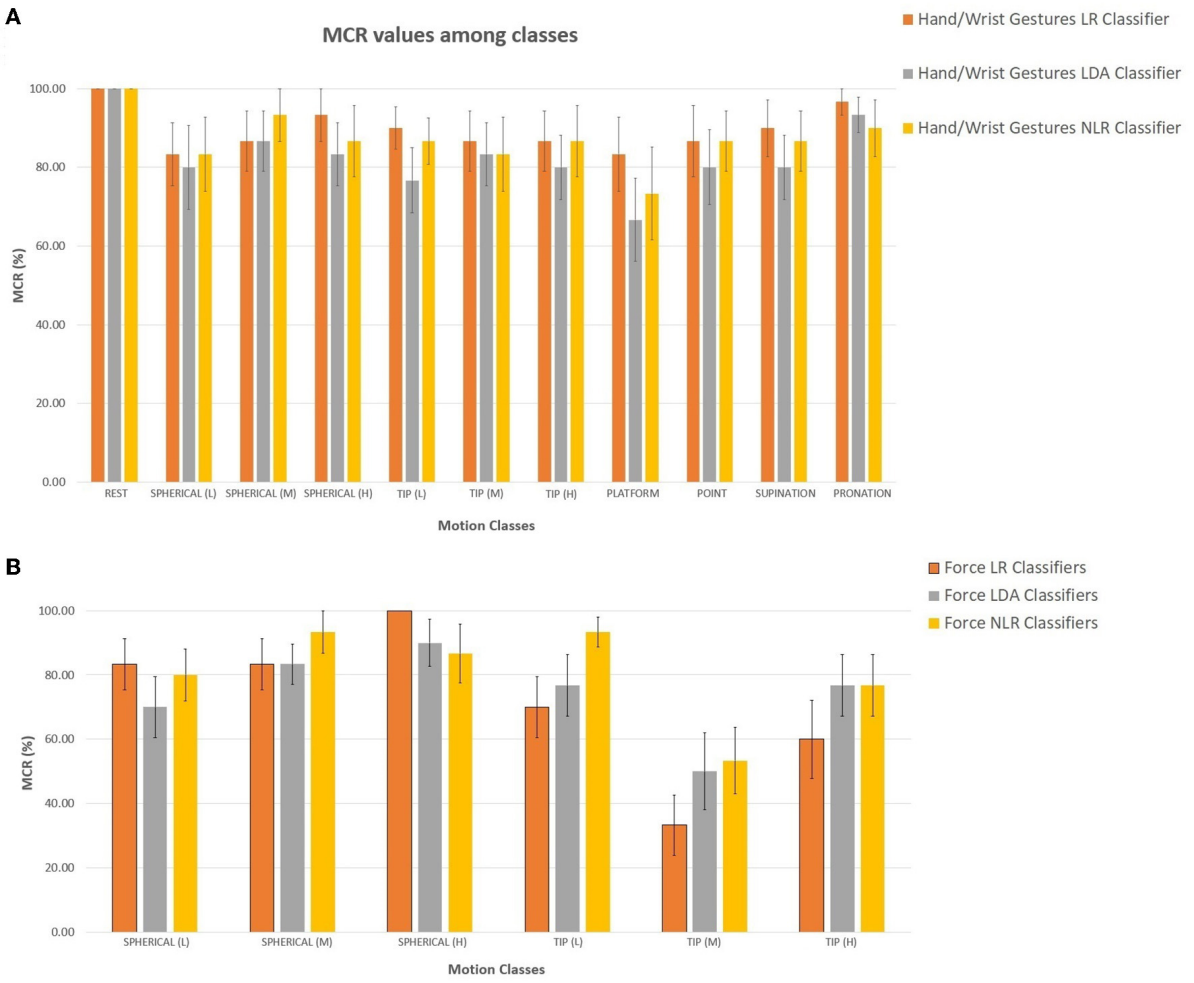
**(A, B)** Average MCR percentages calculated for 15 transradial amputees using **(A)** the LR (in orange), LDA (in gray), and NLR (in yellow) “hand/wrist gestures classifier” **(A)** and the LR (in orange), LDA (in gray), and NLR (in yellow) force classifiers **(B)**: the “spherical force classifier” and the “tip force classifier” for the spherical and tip grasp, respectively. The mean MCR percentage was calculated over three repetitions of the reported motion classes.

The motion completion rate (MCR) (Kuiken et al., 2009) was used as a performance indicator: MCR indicated the percentage of successfully completed motions out of the total attempted motions. All three classifiers with FE (“hand/wrist gestures classifier”, “spherical force classifier”, and “tip force classifier”) were tested in real-time with the LR, LDA, and NLR algorithms and produced a new prediction every 90 ms. In particular, the online accuracy and the MCR are reported in Figures 5, 6 and calculated over three repetitions of all the 11 motion classes (in this case, the three force levels for the spherical and tip classes were considered as a motion class).

For the “hand/wrist gestures classifier”, the real-time mean accuracy among motion classes was equal to 93.12 ± 1.18, 91.24 ± 1.25, and 92.06 ± 1.19 for the LR, LDA, and NLR classifiers, respectively. In detail, for the “hand/wrist gestures classifier”, the spherical class with low force and the tip class with high force showed the lowest accuracy values (below 90%) for the three algorithms. Regarding the force level classification, the medium force, related to the “spherical force classifier”, and the low force, related to the “tip force classifier”, obtained below-average (90%) accuracy values. The mean MCR values are reported in Figure 6. In detail, for the “hand/wrist gestures classifier”, the lowest MCR values (below 90%) for the spherical with low force, the tip with high force, and the platform motion classes were obtained. For the force classifiers, the spherical with medium force and the tip with low and medium force reached the minimum MCR values. The *U*-test with Bonferroni correction (*P* < 0.016) applied to the online accuracy and the MCR values pointed out no statistically significant difference among the three algorithms.

**Figure 7:**
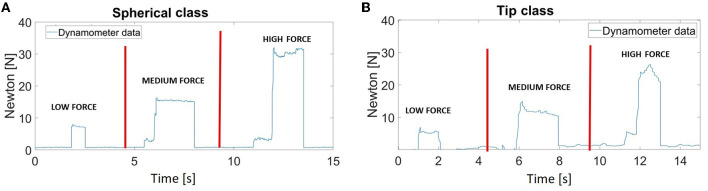
The dynamometer data showed the force signals obtained when the prosthesis applied the three force levels (low, medium, and high) during the spherical **(A)** and tip class **(B)**.

Finally, Figure 7 shows the signals obtained from the dynamometer when the amputee performed grasping tasks (spherical or tip grasp) with the prosthesis. Two outputs, related to the desired motion class and the corresponding force level in the case of a grasping task, were sent simultaneously to the prosthesis. In detail, the “hand/wrist gestures classifier” discriminated the desired motion class to perform with the prosthesis: if the predicted output was the “spherical” or “tip” class, the force levels information was also added and the classification approach become hierarchical. The effect of force level classification was translated into the control of the hand device at three different speeds, representing each force level. Thus, Figures 7A, B show the force signals obtained when the prosthesis applied the three force levels (low, medium, and high) on the dynamometer during the “spherical” and “tip” class, respectively.

## 4. Discussion

In this study, a hierarchical PR-based control that allows the recognition of seven gestures and, for grasping tasks, three levels of force, was presented. This approach, based on the strategy presented previously by Leone et al. (2019), was successfully used to control a prosthetic system. Both the offline and real-time performance were evaluated for 15 transradial amputees. A statistical analysis, based on the *U*-test with Bonferroni correction, was conducted to assess the effect of the FE on the F1 score values for three algorithms: LR, NLR, and LDA. The F1 score and classification accuracy for the offline evaluation were reported along with the real-time accuracy and MCR values for the online evaluation.

### 4.1. Offline performance

The main results of the preliminary analysis showed a statistically significant difference related to F1 score improvements when considering the FE. This effect can be summarized as a 10.42% increase of the mean F1 score values of the seven output classes and a 4.84% reduction of the misclassification error values. These results seem to be very promising thanks to the simultaneous classification of hand/wrist gestures and force levels by transradial amputees with an F1 score of more than 90% for the “hand/wrist gestures classifier” and 95% for the force classifiers, implemented with the three algorithms. The performance obtained with 31 healthy subjects (Leone et al., 2019) were compared with the results of this study. For the NLR and LDA algorithms, the mean F1 score values were approximately 95% for the “hand/wrist gestures classifier” and more than 97% for the force classifiers, except for the NLR “tip force classifier”, which had a mean F1 score value of 94%. In this study, amputees had mean F1 score values that were 4 and 2% lower than those of the healthy subjects for the NLR and LDA “hand/wrist gestures classifier” and “spherical force classifier”, respectively, while they obtained 1% higher mean F1 score values than those of healthy subjects for the NLR “tip force classifier”. Additionally, the offline misclassification error rates, defined as the percentage of incorrect classifications, were used to evaluate the classifier performance: they remained within 10% and this can be considered a positive result for a usable system (Scheme and Englehart, 2011). The confusion matrices, available in the Supplementary material, confirmed the good results in terms of accuracy. For the force classifiers, the low and medium force levels produced most of the misclassified data out of the main diagonal. Some amputees (as the subjects P1, P2, P3, P5, P7, and P12, described in Supplementary Table 1) found modulating between low and medium force levels more difficult than others, especially during a tip grasp. This finding may be due to the shorter length of the stump. In addition, the dynamometer data (Figure 7) confirmed that the prosthesis applied the three levels of force.

### 4.2. Real-time performance

The real-time results of the hierarchical PR-based strategy were reported. For each algorithm (i.e., LR, LDA, and NLR), the online accuracy and the MCR were reported to identify the robustness of the proposed system.

Compared with the literature, the proposed PR-based hierarchical classification strategy of hand/wrist gestures and force levels was tested on 15 transradial amputees instead of three (Fang et al., 2022). In addition, the online accuracy for 11 motion classes was evaluated. Comparing the online accuracy for the wrap motion (comparable with the presented spherical gesture) and two-finger pinch gestures (comparable with the tip motion class), these values did not exceed 90% for the three force levels in Fang et al. (2022). In this study, the medium and high force levels of the spherical motion class and the high level of the tip motion achieved the highest online accuracy values (above 90%). For the force classifiers, the obtained MCR values may depend on the difficulty of amputees to keep the muscle contraction stable for both the grasping task and force levels. In particular, the tip grasping task with the medium level was the most difficult to reproduce and keep stable in real-time for the majority of amputees.

Moreover, in Farrell and Weir (2007), the classification outputs for the final decision were made within 300 ms from the end of the desired motion. In this study, both the LR and NLR guaranteed a response time of 100 ms, in real-time, which is lower than the delay of 160 ms reported by Fang et al. (2022).

This study made it possible to evaluate the performance of the proposed strategy based on the approach presented by Leone et al. (2019), both offline and for real-time applications, and investigated a new methodology for the perception of the force applied by the patient. The solution with the best performance could be obtained using the LR algorithm with FE for the “hand/wrist gestures classifier” and the NLR with FE for the spherical and tip force classifiers. However, a more in-depth analysis should provide more evidence for this supposition.

## 5. Conclusion

This study presented a hierarchical classification approach that is able to discriminate hand/wrist gestures and force levels on a prosthetic device. In a previous study by Leone et al. (2019), only the offline performance of this classification strategy was evaluated on 31 healthy subjects. In this paper, the offline performance of amputees was promising and similar to that of healthy subjects: the 15 transradial amputees had an F1 score of more than 90% for the “hand/wrist gestures classifier” and 95% for the force classifiers, implemented with the three algorithms with FE. Additionally, the offline misclassification error rates remained within 10% and this can be considered a positive result for a usable system (Scheme and Englehart, 2011), and compared with the literature (Fang et al., 2022). These results demonstrate the feasibility of the hierarchical PR-based approach to simultaneously control hand/wrist gestures and force levels, particularly when considering amputee subjects. Moreover, a response time of 100 ms, with a mean online accuracy above 90%, makes it suitable for real-time applications. Future studies will be focused on the validation of the presented method on an embedding solution of this classification system: the final electronic device, composed of a microcontroller unit, will allow amputees to wear the prosthetic device to simultaneously control hand/wrist gestures and force levels.

## Data availability statement

The original contributions presented in the study are included in the article/Supplementary material, further inquiries can be directed to the corresponding author.

## Ethics statement

The studies involving human participants were reviewed and approved by Comitato Etico di Area Vasta Emilia Centro; protocol number: CP-PPRAS1/1-01; date of approval: 04/10/2018. The patients/participants provided their written informed consent to participate in this study.

## Author contributions

FL analyzed the literature, designed the proposed approach, acquired and analyzed the experimental data, and wrote the paper. FM analyzed the literature, designed the proposed approach, designed the prosthetic setup, acquired the data, and wrote the paper. CG contributed to the design of the proposed approach, to the analysis of the experimental data, and wrote the paper. FC contributed to the design of the proposed approach, wrote the paper, and supervised the study. EG contributed to the design of the proposed approach and supervised the study. LZ designed the paper and supervised the study. All authors read and approved the manuscript.

## Footnotes

^1^Comitato Etico di Area Vasta Emilia Centro; protocol number: CP-PPRAS1/1-01; date of approval: 04/10/2018.
