# Supplementary material for: Hierarchical strategy for sEMG classification of the hand/wrist gestures and forces of transradial amputees
Source: Front Neurorobot. 2023 Mar 9;17:1092006. doi: 10.3389/fnbot.2023.1092006 (PMC10035594; doi:10.3389/fnbot.2023.1092006)
Supplement: Supplementary file 1 [file Presentation_1.pdf]

Supplementary Material

1 FEATURES EXTRACTION

The EMAV and EWL were employed instead of the MAV and WL, respectively, because the combination of them with other time domain features has been considered valuable for performance enhancement in rehabilitation and clinical applications Too et al. (2019). The EMAV is an extension of the MAV, defined as the sum of the absolute value of EMG signals Purushothaman and Vikas (2018) and can be calculated as Eq.S1:

$$EMAV_i = \frac{1}{L}\sum_{i=1}^{L} |(x_i)^p| \tag{S1}$$

where x_i is the ith time sample in a window and by L the total length of the window and the parameter p, defined as :

$$p = \begin{cases} 0.75, & if\ i\ \geq\ 0.2\ L\ \&\ \ i\ \leq\ 0.8\ L \\ 0.50, otherwise \end{cases} \tag{S2}$$

is used in the Eq.S1 and Eq.S3 to identify the influence of the sample within the signal (range 20÷80%, Too et al. (2019)).

The EWL is an extension of WL and represents the cumulative length of the EMG signal waveform and can be calculated as Eq.S3:

$$EWL = \sum_{i=2}^{L} |(x_i - x_{i-1})^p| \tag{S3}$$

where the parameter p is defined as:

$$p = \begin{cases} 0.75, & if\ i\ \geq\ 0.2\ L\ \&\ \ i\ \leq\ 0.8\ L \\ 0.50, otherwise \end{cases} \tag{S4}$$

The Slope Sign Change represents the frequency information of the sEMG signal and measures the changes in the slope sign of the sEMG signal and counts them. It is defined as Eq.S5:

$$SSC = \frac{1}{L}\sum_{i=2}^{L-1} f[(x_i - x_{i-1}) \times (x_i - x_{i+1})] \tag{S5}$$

where $f(x_i)$ is defined as:

$$f(x_i) = \begin{cases} 1, & x \geq threshold \\ 0, & otherwise \end{cases} \tag{S6}$$

The Root Mean Square is the mean power of the signal and is defined as Eq.S7:

$$RMS = \sqrt{\frac{1}{L}\sum_{i=1}^{L} x_i^2} \tag{S7}$$

The Variance represents a statistical measure of how the signal varies from its average value and is defined as Eq.S8:

$$VAR = \frac{1}{L-1}\sum_{i=1}^{L} x_i^2 \tag{S8}$$

2 LOGISTIC REGRESSION (LR) AND NON-LINEAR LOGISTIC REGRESSION (NLR) CLASSIFIERS

The following cross-entropy error cost function was introduced with regularization term to improve the generalization performance on unseen data Eq.S9

$$\begin{aligned} J(\theta,\theta_0) = \sum_{i=1}^{n} -y^{(i)} \cdot \ln g(\theta^T \cdot x^{(i)} + \theta_0) - (1 - y^{(i)}) \cdot \\ \cdot \ln(1 - g(\theta^T \cdot x^{(i)} + \theta_0)) + \tfrac{\lambda}{2}\|\theta\|^2 \end{aligned} \tag{S9}$$

where y is the known class membership of the $i-th$ sample, and λ is the regularization parameter that adds a penalty on the cost function when the magnitudes of the fitting parameters increase. The gradient of the cost function is a vector where the J(θ) element is defined as Eq.S10:

$$\begin{cases} \frac{\partial J(\theta)}{\partial \theta_0} = \frac{1}{m}\sum_{i=1}^{m}(h_\theta(x^{(i)}) - y^{(i)})x_j^{(i)} & \text{for j=0} \\ \frac{\partial J(\theta)}{\partial \theta_0} = \frac{1}{m}\sum_{i=1}^{m}(h_\theta(x^{(i)}) - y^{(i)})x_j^{(i)} + \frac{\lambda}{m}\theta_j & \text{for j}\geq 0 \end{cases} \tag{S10}$$

where $h_\theta(x)$=θ_0+θ_1*x+θ_2*x^2+...$\theta_i x^i$ is the hypothesis function and m is the number of samples used to train the algorithm. The first-order iterative optimization algorithm "Gradient descent" was used for finding a local minimum of the multivariate differentiable cost function, with a maximum number of iterations equals to 150 Cheikh et al. (2016). In detail, the Polack-Ribiere flavour of conjugate gradients was used to compute search directions, and a line search with quadratic and cubic polynomial approximations and the Wolfe-Powell stopping criteria was employed together with the slope ratio method for guessing initial step sizes Borgul et al. (2012).

The thresholds tested (TH) range from 0.2 to 0.8 with step to 0.01. In detail, the prediction of class labels h_θ for the LR algorithm was achieved by comparing the probability distribution $P(y|x)$ with a decision threshold (TH) as in Eq.S11.

$$h_\theta = \begin{cases} P(1 \mid x,\theta) \geq TH \rightarrow 1 \\ P(1 \mid x,\theta) < TH \rightarrow 0 \end{cases} \tag{S11}$$

3 LINEAR DISCRIMINANT ANALYSIS

The training of the classifiers was performed by using Eq.S12, and Eq.S13. In detail, the following decision function (Eq.S12) was used to discriminate between two different classes and to assign class label 1 or 2 to unknown data:

$$h_\beta(x) = \begin{cases} (\beta^T \cdot x + \beta_0) \geq 0 \rightarrow 1 \\ (\beta^T \cdot x + \beta_0) < 0 \rightarrow 2 \end{cases} \tag{S12}$$

where β and β_0 are, respectively, the classification parameters vector and the bias term ((Eq.S13)).

$$\begin{cases} \beta = \Sigma^{-1} \cdot (\mu_1 - \mu_2) \\ \beta_0 = -\beta^T \cdot \left(\frac{\mu_1+\mu_2}{2}\right) + \ln\left(\frac{\Pi_1}{\Pi_2}\right) \end{cases} \tag{S13}$$

where Σ is the pooled covariance matrix, μ_1, μ_2, Π_1, Π_2 are the mean vectors, and the prior probabilities of class 1 and class 2.

4 SUPPLEMENTARY TABLES AND FIGURES

Table S1. Summary of the trans-radial patients' information

Patient	Age	Sex	Amputatee side	Dominant limb	Years from amputation	Expert	Stump length (cm)
P1	25	M	DX	DX	11	yes	12
P2	37	M	SX	DX	13	yes	9
P3	52	F	DX	DX	37	no	6
P4	57	M	DX	DX	25	yes	15
P5	40	F	SX	DX	26	yes	8
P6	54	M	DX	DX	24	yes	14
P7	26	F	SX	DX	25	no	7
P8	61	M	DX	DX	13	yes	22
P9	32	M	DX	DX	6	yes	7
P10	43	M	DX	DX	18	yes	21
P11	64	M	SX	DX	20	yes	7
P12	31	M	DX	DX	7	yes	5
P13	41	M	DX	DX	5	yes	23
P14	47	M	SX	DX	25	yes	26
P15	65	M	DX	DX	50	yes	14.5

Table S2. Mean value and standard deviation of F1Score and Accuracy (%) with FE of the Hand/Wrist gestures Classifier calculated for 15 trans-radial amputees

HAND/WRIST GESTURE CLASSIFIER

	**LR**		**LDA**		**NLR**	
	F1 (%)	Accuracy (%)	F1 (%)	Accuracy (%)	F1 (%)	Accuracy (%)
Rest	94.38 ± 2.20	100.00 ± 0.00	94.16 ± 2.32	100.00 ± 0.00	94.14 ± 2.24	100.00 ± 0.00
Spherical	91.01 ± 2.78	87.54 ± 3.80	88.21 ± 4.37	85.08 ± 5.88	88.51 ± 3.61	85.96 ± 4.50
Tip	89.18 ± 2.03	87.89 ± 2.68	88.61 ± 2.61	87.01 ± 3.01	88.31 ± 2.01	87.19 ± 2.54
Open	90.67 ± 2.81	90.35 ± 3.19	91.99 ± 2.67	92.45 ± 2.54	91.25 ± 2.33	91.05 ± 2.68
Point	90.47 ± 3.21	91.579 ± 3.36	92.88 ± 2.37	94.73 ± 2.24	89.86 ± 2.51	90.87 ± 2.72
Supination	91.69 ± 3.15	90.17 ± 4.13	92.57 ± 3.05	91.40 ± 3.36	89.75 ± 3.60	87.89 ± 4.30
Pronation	94.08 ± 2.41	94.21 ± 2.71	95.10 ± 2.35	94.03 ± 3.20	92.13 ± 2.21	91.93 ± 2.66
**MEAN**	**91.65 ± 2.66**	**91.68 ± 2.84**	**91.93 ± 2.82**	**92.11 ± 2.89**	**90.57 ± 2.64**	**90.70 ± 2.77**

Table S3. Mean value and standard deviation of F1Score and Accuracy (%) with FE of the Spherical force Classifier and Tip force classifier calculated for 15 trans-radial amputees

SPHERICAL FORCE CLASSIFIER

	LR		LDA		NLR	
	F1Score	Accuracy (%)	F1Score	Accuracy (%)	F1Score	Accuracy (%)
LOW	96.19 ± 2.06	97.44 ± 1.23	98.27 ± 0.87	98.97 ± 0.70	93.03 ± 3.86	93.85 ± 4.76
MEDIUM	94.34 ± 3.16	92.31 ± 4.50	97.89 ± 1.09	97.44 ± 1.44	93.61 ± 2.97	93.33 ± 3.59
HIGH	99.26 ± 0.53	99.49 ± 0.51	99.73 ± 0.27	99.49 ± 0.51	99.04 ± 0.72	99.49 ± 0.51
**MEAN**	**96.60 ± 1.92**	**96.41 ± 2.08**	**98.63 ± 0.74**	**98.63 ± 0.88**	**95.23 ± 2.52**	**95.56 ± 2.95**

TIP FORCE CLASSIFIER

LOW	94.00 ± 4.20	94.36 ± 5.12	95.33 ± 1.71	96.41 ± 1.66	94.59 ± 2.69	96.41 ± 2.59
MEDIUM	95.80 ± 2.12	95.90 ± 2.59	94.21 ± 2.20	94.36 ± 2.54	94.24 ± 3.18	92.31 ± 3.75
HIGH	99.28 ± 0.52	100.00 ± 0.00	98.40 ± 0.86	97.44 ± 1.23	99.01 ± 0.67	99.49 ± 0.51
**MEAN**	**96.36 ± 2.28**	**96.75 ± 2.57**	**95.98 ± 1.59**	**96.07 ± 1.81**	**95.95 ± 2.18**	**96.07 ± 2.28**

The *Fig.*S1 showed the average confusion matrix when testing the LR, LDA, and NLR “Hand/wrist gestures classifier”, “Spherical force classifier” and “Tip force classifier” (A, B, C, respectively) on the Test Set.

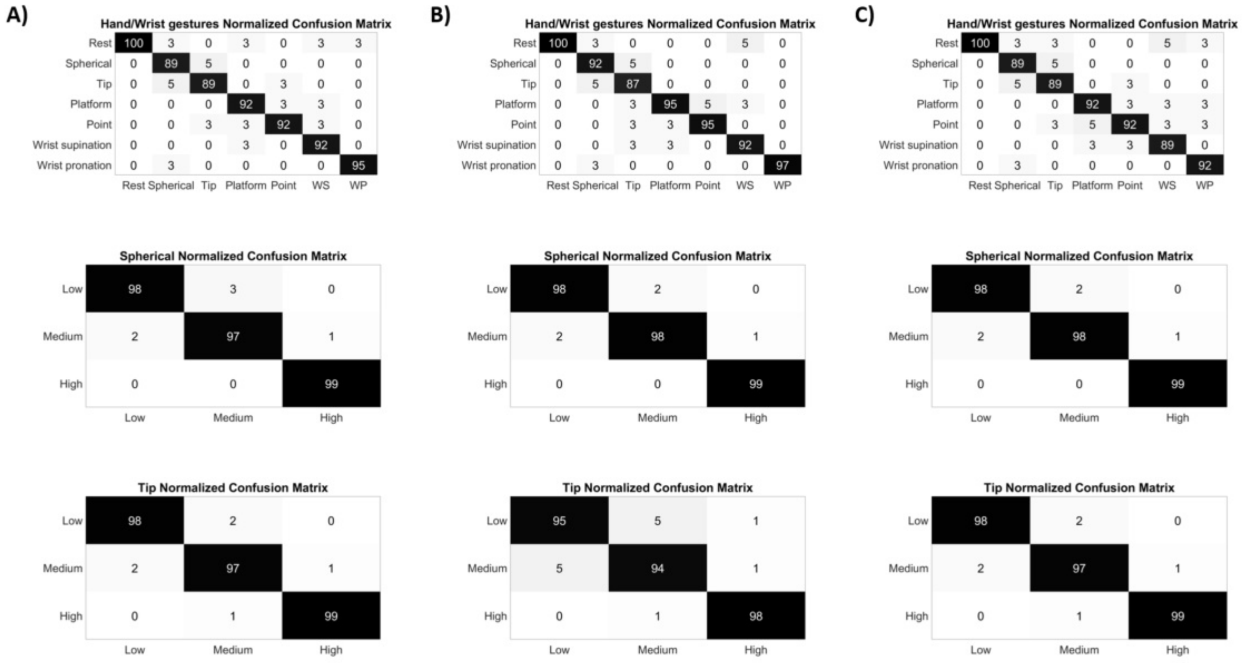

Figure S1. Normalized confusion matrix of the “Hand/wrist gestures classifier”, “Spherical force classifier” and “Tip force classifier” obtained with LR algorithm with FE (A) and LDA algorithm with FE (B) and NLR algorithm with FE (C). The confusion matrices are normalized with respect to the number of data belonging to the “TS”. On the main diagonal the cardinality of the correct classifications is reported; for the “Hand/wrist gestures classifier”, the cardinality of the misclassified data related to the 7 output classes representing the hand gestures is reported. While for the “Spherical force classifier” and “Tip force classifier”, the cardinality of the misclassified data related to the 3 output classes that represented the force levels (Low, Medium, High) are reported.

REFERENCES

Borgul, A., Margun, A., Zimenko, K., Kremlev, A., and Krasnov, A. (2012). Intuitive control for robotic rehabilitation devices by human-machine interface with emg and eeg signals. In *2012 17th International Conference on Methods & Models in Automation & Robotics (MMAR)* (IEEE), 308–311

Cheikh, A. B., Ayari, M., Langar, R., and Saidane, L. A. (2016). Ohdp: Optimized handover with direction prediction scheme using linear regression for femtocell networks. In *2016 International Conference on Performance Evaluation and Modeling in Wired and Wireless Networks (PEMWN)* (IEEE), 1–6

Purushothaman, G. and Vikas, R. (2018). Identification of a feature selection based pattern recognition scheme for finger movement recognition from multichannel emg signals. *Australasian physical & engineering sciences in medicine* 41, 549–559

Too, J., Abdullah, A. R., and Saad, N. M. (2019). Classification of hand movements based on discrete wavelet transform and enhanced feature extraction. *Int. J. Adv. Comput. Sci. Appl* 10, 83–89
